# Epic Allies: Development of a Gaming App to Improve Antiretroviral Therapy Adherence Among Young HIV-Positive Men Who Have Sex With Men

**DOI:** 10.2196/games.5687

**Published:** 2016-05-13

**Authors:** Sara LeGrand, Kathryn Elizabeth Muessig, Tobias McNulty, Karina Soni, Kelly Knudtson, Alex Lemann, Nkechinyere Nwoko, Lisa B Hightow-Weidman

**Affiliations:** ^1^ Center for Health Policy and Inequalities Research Duke Global Health Institute Duke University Durham, NC United States; ^2^ Department of Health Behavior University of North Carolina at Chapel Hill Chapel Hill, NC United States; ^3^ Caktus Consulting Group, LLC Durham, NC United States; ^4^ Department of Medicine University of North Carolina at Chapel Hill Chapel Hill, NC United States

**Keywords:** mobile applications, video games, serious games, HIV, medication adherence, health knowledge, attitudes, practice, youth, men who have sex with men

## Abstract

**Background:**

In the United States, the human immunodeficiency virus (HIV) disproportionately affects young men who have sex with men (YMSM). For HIV-positive individuals, adherence to antiretroviral therapy (ART) is critical for achieving optimal health outcomes and reducing secondary transmission of HIV. However, YMSM often struggle with ART adherence. Novel mobile phone apps that incorporate game-based mechanics and social networking elements represent a promising intervention approach for improving ART adherence among YMSM.

**Objective:**

This study used a multiphase, iterative development process to create an ART adherence app for YMSM.

**Methods:**

The three-phase development process included: (1) theory-based concept development jointly by public health researchers and the technology team, (2) assessment of the target population’s ART adherence needs and app preferences and development and testing of a clickable app prototype, and (3) development and usability testing of the final app prototype.

**Results:**

The initial theory-based app concept developed in Phase One included medication reminders, daily ART adherence tracking and visualization, ART educational modules, limited virtual interactions with other app users, and gamification elements. In Phase Two, adherence needs, including those related to information, motivation, and behavioral skills, were identified. Participants expressed preferences for an ART adherence app that was informational, interactive, social, and customizable. Based on the findings from Phase Two, additional gaming features were added in Phase Three, including an interactive battle, superhero app theme, and app storyline. Other features were modified to increase interactivity and customization options and integrate the game theme. During usability testing of the final prototype, participants were able to understand and navigate the app successfully and rated the app favorably.

**Conclusions:**

An iterative development process was critical for the development of an ART adherence game app that was viewed as highly acceptable, relevant, and useful by YMSM.

## Introduction

In the United States, youth accounted for approximately 26% of new human immunodeficiency virus (HIV) infections in 2010 [1]. Among youth, young men who have sex with men (YMSM) accounted for 72% of new HIV infections and were the only risk group that experienced a significant increase in HIV incidence [1,2]. Once diagnosed with HIV, youth are less likely to engage in HIV care, receive a prescription for antiretroviral therapy (ART), and have sustained adherence [3]. For those on ART, daily, lifelong adherence is necessary to maximize health benefits and reduce the likelihood of onward HIV transmission [4-8]. Novel, sustainable interventions that improve adherence to ART among HIV-positive YMSM are needed to improve individual health, decrease health care costs, and reduce HIV transmission risk [8].

The near saturation of the smartphone market among youth and young adults in the United States has created opportunities for reaching a large number of young people with health behavior interventions, including those that address ART adherence [9]. In a recent review of the literature on smartphone, Internet, and Web 2.0 interventions for HIV prevention and care, 10 published or ongoing studies were identified that used smartphones to improve ART adherence, including the intervention described in this paper, Epic Allies. Epic Allies was the only intervention identified that was explicitly designed to meet the specific adherence needs of YMSM [10].

Games are increasingly used to address behavioral and psychological factors associated with adherence to medical treatment regimens [11]. Games are goal-oriented, immersive, challenging, and motivating and can be used to improve attitudes and self-efficacy for health behavior change [12-15]. Social engagement and provision of support are also powerful tools for behavior change, particularly for HIV-positive YMSM who often experience social isolation from HIV-related stigma and homophobia [16-18]. Social networking is one tool that can be used to connect individuals around a specific health issue and allow for the provision and receipt of social support [19]. Because youth and young adults are the most avid users of games and social media [20,21], inclusion of gaming and social networking-based elements into mobile phone applications represents a promising health behavior change intervention strategy. The purpose of this study was to develop Epic Allies, a gaming app with behavior tracking features and social networking elements, to improve ART adherence among HIV-positive YMSM. This paper highlights the importance of using an iterative design process, including obtaining feedback from YMSM at each stage of development, to achieve optimal app design and functionality.

## Methods

### Introduction

The Epic Allies development team was assembled in September 2013. The team consisted of medical and public health researchers from University of North Carolina (UNC) and Duke University (Duke) and a technology team from Caktus Consulting Group (Caktus) with expertise in app design, development, and programming. During the initial meeting, the team agreed to a three-phase approach to app development. The first phase included the development of the initial app concept, which was theoretically grounded in the Information, Motivation, and Behavioral Skills (IMB) model of behavior change (September 2013) [22]. The second phase involved the development and refinement of an early prototype of selected app features and collection of formative data from HIV-positive YMSM, ages 18 to 29, to inform app development (October-December 2013). The third phase consisted of final app prototype development and internal and external usability testing (January-May 2014).

### Phase One

Weekly team meetings focused on app concept development based on the IMB model. The IMB, which has frequently been used to guide the development of ART adherence interventions, conceptualizes health behavior change (eg, medication adherence) as a product of mediators including information about the behavior, motivation to change, and the skills needed to achieve change [22]. Using this model as a guide, our multidisciplinary team outlined and iteratively created a paper prototype of potential features, including gaming and social networking elements.

### Phase Two

At the beginning of Phase Two, a clickable prototype of the Epic Allies app was developed for the Android operating system. Three focus groups were then conducted in Raleigh, Durham, and Charlotte, North Carolina with 20 HIV-positive YMSM, ages 20 to 28 to (1) assess ART adherence information, motivation, and behavioral skills needs, (2) determine strategies to address these needs via a mobile app, and (3) gather feedback on the evolving features of Epic Allies and future feature concepts. Before the focus groups began, each participant completed a brief survey on sociodemographics, Internet/mobile phone use, and ART adherence and selected a pseudonym to protect participant confidentiality during the focus group. After discussing adherence needs and strategies for addressing them through an app, participants used study smartphones to explore and provide feedback on the current Epic Allies prototype. The duration of the focus groups was approximately 90 minutes. Groups were audio recorded, transcribed, and then analyzed using Dedoose qualitative data analysis software [23] to identify a range of themes across participants. Quotes representing common responses and variations within each theme were identified by study team consensus. After each focus group, the study team met to discuss preliminary findings and themes and to identify key features to add or remove from the Epic Allies prototype. An updated clickable prototype was created prior to each subsequent focus group in an iterative development process.

### Phase Three

The team worked collaboratively to modify the initial conceptual design of the app based on the focus group findings and created a final plan for app features and functionality. The final app prototype was developed incrementally with components added and refined weekly. Research staff members from UNC and Duke conducted ongoing testing of new versions of the prototype to identify bugs and provide feedback on the user interface and navigation experience. After a full prototype was completed, five new internal testers assessed all functionality of the app to identify any remaining technical glitches or usability concerns. Caktus used the detailed feedback from this internal testing to develop a polished version of the final app prototype for external usability testing.

In April and May 2014, external usability testing was conducted with seven HIV-positive YMSM, ages 20 to 28. External usability testing aimed to assess whether users: could successfully navigate features and functions of the app, could comprehend the educational content, and found the app to be engaging and relevant. After providing informed consent and completing a brief survey on sociodemographics, Internet/mobile phone use, and ART adherence, participants met with a member of the development team and a member of the research team to “walk through” the app. Other members of the team (including developers and researchers) watched a live audio-video stream of the testing in a separate room. To protect participant confidentiality during the audio-video streaming, participants used pseudonyms and the video only captured the participant’s hand movements and the app screen. Participants were given a checklist of tasks to complete within the app at hypothetical intervention days 1, 21, and 42. These days were selected to give usability participants an opportunity to test features they would see on day 1, representing the first time they had used the app, and on days 21 and 42 after they had been tracking their adherence and gaining power and points within the game. A checklist was provided to guide users through the tasks for each day. Participants were asked to “think aloud” and share thoughts and questions that came up as they were completing tasks. After completing the tasks and providing feedback, participants completed a 9-question survey adapted from the System Usability Scale [24-27]. Usability testing sessions were recorded, transcribed and analyzed using the same methods described for the focus groups. Quantitative survey and usability results were summarized using Excel.

## Results

### Phase One

The initial Epic Allies app concept included features designed to address potential IMB ART adherence needs of HIV-positive YMSM based on clinical expertise and a review of the literature. The initial proposed features and their relationship to the IMB are highlighted in Table 1.

### Phase Two

#### Descriptive Statistics

The mean age of focus group participants (n=20) was 24 years. All participants identified as black or African American. Most (15/19, 79%) earned less than US$20,999 per year. All participants owned a smartphone (20/20, 100%) and (11/20, 55%) had used a health app in the past 3 months. There was substantial variation in medication adherence patterns among the 19 participants currently on ART: five had missed at least one dose in the past week, five had missed at least one dose in the past month, and four reported never missing any doses. Key focus group findings are organized into three sections: medication adherence information, motivation, and behavioral skills needs; strategies for addressing medication adherence information, motivation, and behavioral skills needs in an app; and strategies to motivate app use.

#### Medication Adherence Information, Motivation, and Behavioral Skills Needs

Information needs identified by participants included understanding dosing times and information about side effects and side effects management. Participants also expressed confusion about appropriate dosing times and the number of pills required per week to constitute medication adherence. In one focus group, participants discussed differences in provider instructions regarding missed doses.

Goku: *And sometimes it’s like my doctor told me that if I miss my time there’s like a 4-hour period or something like that, so I just try to catch it in between.*

Grayland: *Goku, it’s strange that you say your doctor gave you a 4-hour time window to take your meds. Mine only gave me like an hour. I mean he [doctor] said if you happen to miss, you have an hour window. If you don’t take it by then you might as well skip it and take it the next day.* [Goku & Grayland, Raleigh]

Side effects knowledge and management was also a consistent theme across focus groups. Participants felt that those new to medications or switching medications should be aware of potential side effects and how to cope with them.

Acceptance of HIV status was identified as a key adherence motivation need. Participants spoke of the denial and internalized stigma they experienced around their diagnosis and the fear they had around living with a highly stigmatized health condition that would require lifelong medical management.

Because it’s not so much ignorance of other people, it’s the ignorance that you have within yourself that you’re battling with and, ya know, learning these new things. You really have to be mentally accepting to what the new reality is. Yeah it’s a hard, ongoing process. It really continues [for me], extremely continues. It is happening now actually.

Ichiban, Durham

Those who had not accepted their status were less motivated to take their medication, as taking their medication was a daily reminder that they had HIV.

Yeah, cause honestly, it was a good few months before I ever took medication. And in that timeframe of diagnosis to taking medication, it was very easy for me to detach. It was very easy for me to say, this is not real, nahhh, whatever. It didn’t become real until I had to take a pill. When you take a pill, it’s real.

Brett, Raleigh

A lot of the people who you say do forget to take their medications, those are the people who have the mind set of they don’t want the disease, or the health condition, to be a banner for them, they don’t want that to be the representation of them.

Crowned, Charlotte

Social support had a strong influence on motivation to adhere to medications. Some of the participants who disclosed their status reported receiving social support that helped them deal with the fear and uncertainty of an HIV diagnosis. The support often motivated individuals to receive HIV care and adhere to medications.

Then I had a moment, I was just like, ‘Oh my god, my mom’s gonna kill me, she’s gonna kick me out of the house.’ My mom calls me on the way home from work. She’s like, ‘So what did the doctor say? Yeah, he said, oh, so you got it [HIV]? Okay, alright, so we’ll call the doctor tomorrow and see if we can get you some pills.’ I was like, thank you mommy.

Brett, Raleigh

However, many participants did not feel they had people that they could talk to about their diagnosis due to both the fear of HIV stigma or previously experienced stigma associated with their sexuality. Individuals who had not disclosed their status to their social circle often relied on social workers, case managers, or therapists for adherence motivation.

Participants who experienced medication side effects also had less motivation and lower self-efficacy in their ability to fully adhere to their medication.

Yeah, it makes me like really don’t want to take the medications because I really don’t want to have to deal with this or if I have to wake up early and I’m still under the medication and I’m still like, I just be drowsy, and like not wanting to. And sometimes like my friends be like you’re so much meaner when you are on your medications cause I’ll be like, don’t want to be bothered sometimes.

Vee, Durham

Finally, some participants had difficulty integrating medication routines into their daily lives. For example, some men had trouble remembering to take their medications.

Well for me, I know how to take my pill. I do, I think with taking a pill, it’s just like anything else. You may forget. You know what I mean? It’s something that you do on a daily basis. But for the most part, you may forget to take a pill, it happens.

Jerry, Durham

#### Strategies for Addressing Medication Adherence Information, Motivation, and Behavioral Skills Needs in an App

After discussing general adherence needs, participants were asked about the ways an app could help them with adherence. Medication reminders were suggested as an important tool for those who had difficulty remembering to take their medications. Participants emphasized the importance of discreet reminders so that their HIV status would not be revealed if someone saw the message on their phone. Several participants felt that it was important to provide other types of information through the app, including current information about HIV.

It needs something that’s going to keep the person’s attention…maybe a motivational update section where people could post things that are going on with HIV advances, technologies, empowerments [affirmations]…like a news feed or some thing that will keep people intrigued…staying updated and abreast with all of the new HIV information…That would keep me interested.

Stew, Raleigh

Participants also suggested that the app could also be used to deliver important information about adherence at critical times, such as starting medications for the first time or switching medications.

[the app] could be like a whole lot of help especially for people who don’t know some of the side effects they might have to the medication. Like the previous medication that I was on, I had to eat a certain amount of calories when I took the medicine and that would help with questions of that nature and all. Because a whole lot of people just take it. They don’t know its rules to really taking it. Some medicine you got to stay hydrated, some medicine you got to eat, so it’s all kinds of different things that I think [the app] should be able to help with.

Orleans, Charlotte

Several men were in favor of developing an app that could be used to connect with others who may be dealing with similar adherence challenges. Many emphasized the importance of maintaining anonymity in these interactions.

I guess where you can interact with the other users…that way you could find people maybe you can talk to and be in confidence. You wouldn’t have to talk to them face to face…but if you’re having a bad day you could maybe post it and somebody might be going through the same thing and you guys could kinda talk cause, I mean you never know what somebody else might be going through that’s going through the same things that might help.

Batman, Durham

#### Strategies to Motivate App Use

Participants were asked about features that would motivate them to use a medication adherence app. Several overarching themes emerged including the importance of creating an app that is interactive, engaging, social, informational, customizable, and personalized. The men noted that these features would help capture their attention, motivate them to use the app regularly, and improve the likelihood of sustained use.

Participants emphasized the importance of interactivity. Games were seen as a good way to increase app use. In addition, many participants thought rewards for activities within the app would be highly motivating and promote ongoing engagement. Customization was important so users could selectively choose the features most relevant for them.

Put it this way: you want people to use the app daily, it needs to be as comfortable for them as possible. They need to be able to do whatever they want to do with it…because it’s their phone, it’s their medication, it’s their health care.

Brett, Raleigh

Avatars were discussed as a way for users to have a virtual representation within the app and were highly acceptable. There were different perspectives on the level of customization needed to sustain interest, but all participants preferred a personalized avatar.

### Phase Three

#### Development of Full Prototype for Usability Testing

**Figure 1:**
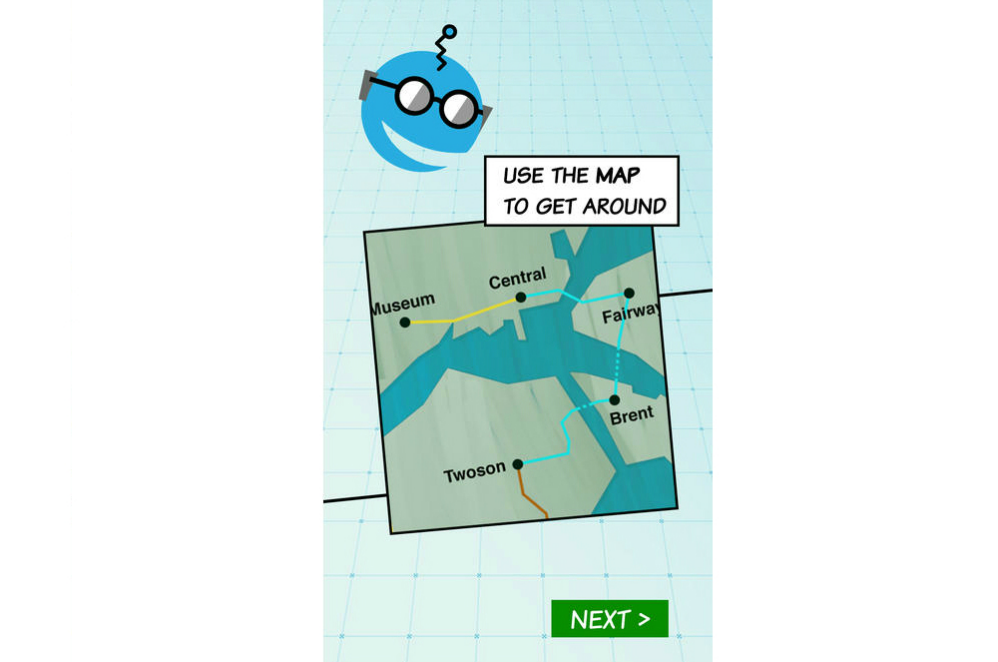
App tutorial.

**Figure 2:**
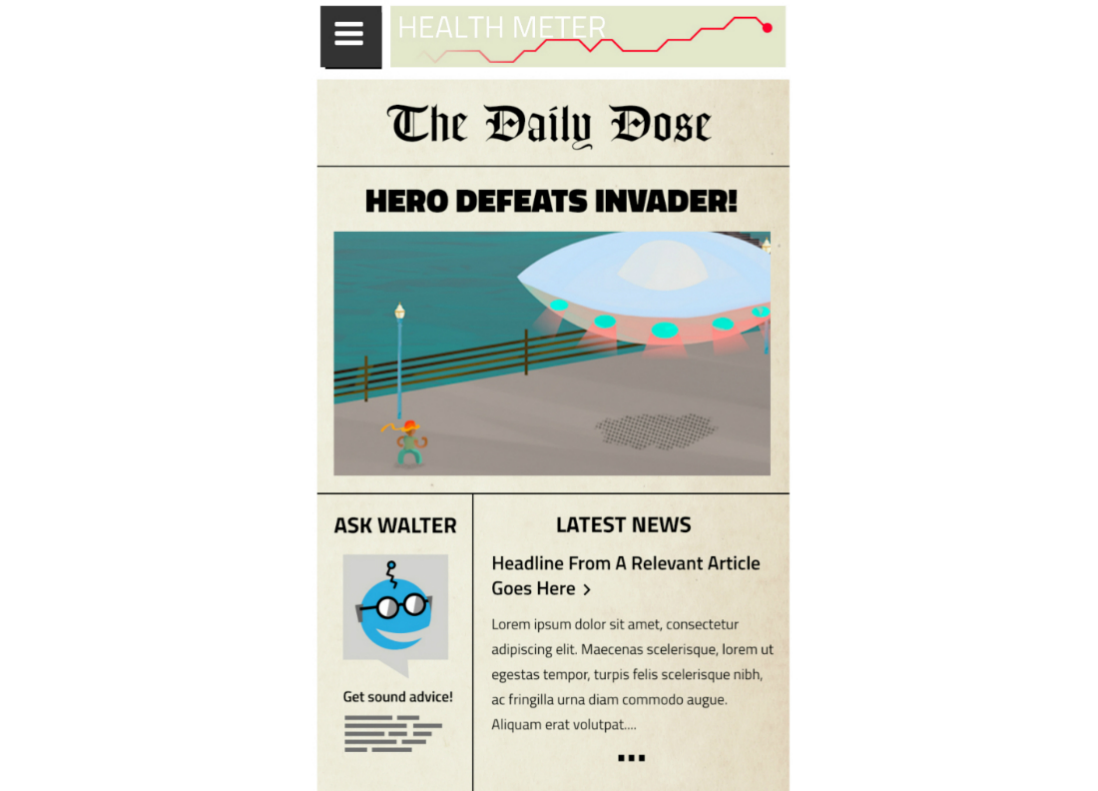
The Daily Dose.

**Figure 3:**
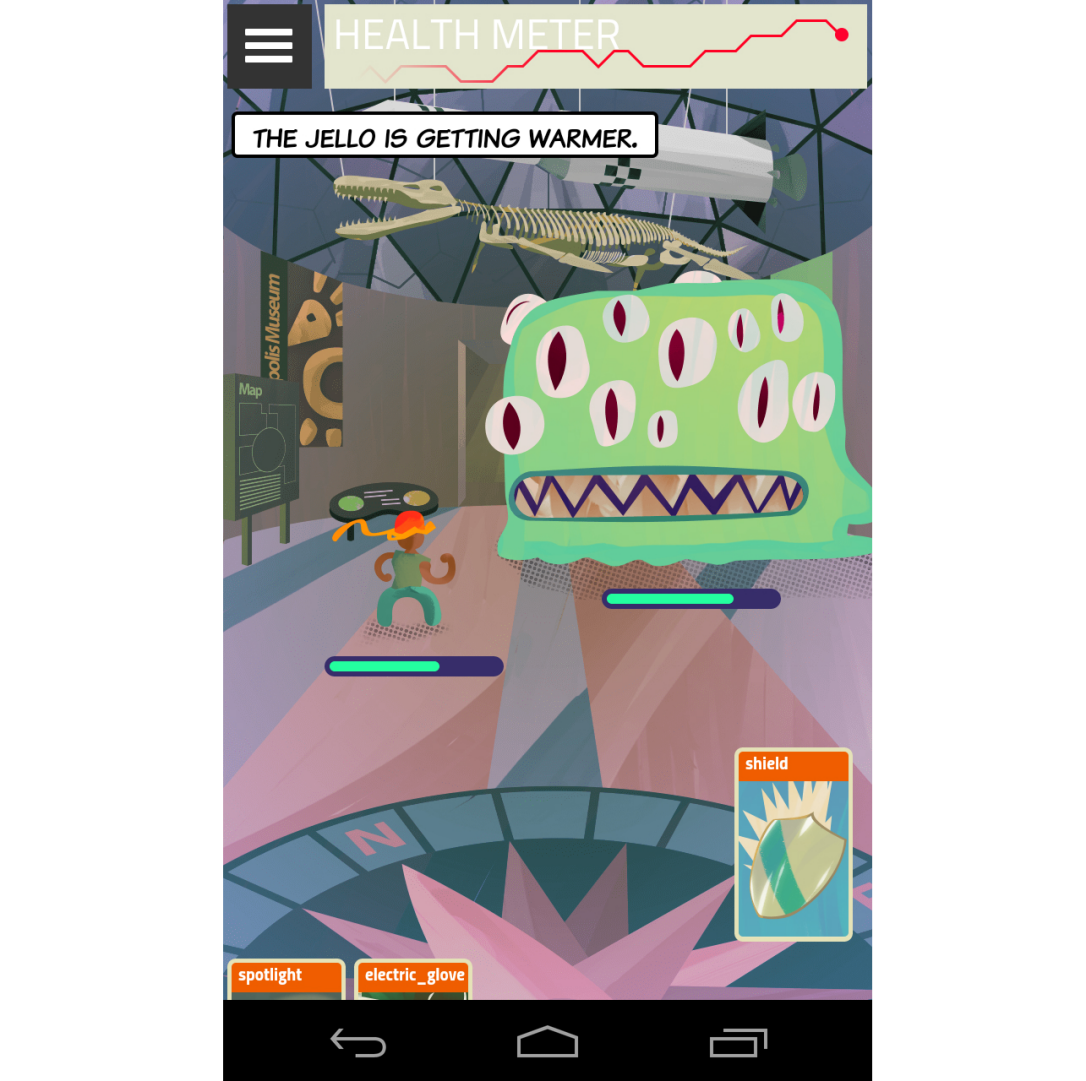
The Battle.

Focus group findings informed the development of the full prototype for usability testing. Changes to the features initially conceptualized in Phase One are highlighted in Table 2. The app storyline and screenshots of selected features are included in Textbox 1 and Figures 1-3.

**Table 1:** Initial proposed features of Epic Allies.

Planned feature	Planned function	Relationship to IMB model
**Customizable avatars**		
	Visual representation of users within the app to facilitate development of an online identity while preserving anonymity to peers.	M^a^: Increases app engagement and facilitates social support.
**Dashboard**		
	Home screen where users enter daily adherence information. Provides a visualization of historical adherence patterns.	I^b^: Tracking and historical visualization of data provides information on adherence behaviors.
		M: Data display visually reinforces positive behaviors and motivates users to change if adherence is suboptimal.
		B^c^: Adherence achievements increase behavior change self-efficacy.
**Reminders and tailored feedback messages**		
	Personalized medication reminders and feedback messages based on users’ successes, setbacks and progress toward their adherence goals.	I: Reminders specify appropriate times to take medications.
		M: Reminders provide a cue to action. Feedback messages affirm positive adherence behaviors and identify adherence challenges.
		B: Reminders help build skills for integrating medication routines in daily life. Feedback messages increase adherence self-efficacy.
**Friends**		
	Users select virtual “friends” to interact with by sending preset messages to challenge, praise or encourage others.	M: Increases social motivation to adhere to medications.
		B: Ally interactions create opportunities for peer modeling and reinforcement of adherence.
**Information modules**		
	Education modules on ART adherence and HIV.	I: Modules provide relevant ART adherence information.
**Gamification**		
	Users earn points for completing selected tasks within the app.	M: Opportunities to earn points increases motivation for app engagement and ART adherence.
		B: Achieving milestones increase adherence self-efficacy.

^a^motivation.

^b^information.

^c^behavioral skills/self-efficacy.

**Table 2:** Changes to Epic Allies for Phase Three

Phase One and Two components	Phase Three components	Phase Three component description	Rationale for change
–	Superhero theme, app storyline, virtual guide	An app storyline was developed to explain the superhero theme, provide a backstory for the app and to introduce users to their role in the game (Textbox 1). A virtual guide, Walter, was created to present the storyline to users and provide a tour of the app.	The storyline and superhero theme were added in response to focus group participants’ request for an engaging and interactive user experience that would motivate regular app use.
–	The Battle	The objective of the Battle is to collect virtual cards used to defeat the monsters in the city of Medopolis. Each day, players fight a new battle to defeat a monster; success or failure depends on the set of three cards they have in their hand. Players can buy and upgrade their cards using points earned by engaging in other parts of the app. Within the Battle, a new social component called the “Spotlight” was created. Using the spotlight, participants can call on their Allies to help defeat difficult monsters.	The Battle feature was introduced to increase app engagement, motivate participants to complete activities within the app, and encourage interactions with Allies.
Customizable avatars	Profile	The avatar concept was expanded to include more detailed information about users such as interests, hobbies, and current ART use.	Focus group participants emphasized the need for social support for adherence. The profile was changed to create a stronger sense of community among users.
Dashboard	Dashboard	The Dashboard feature expanded to include additional tracking options such as exercise, smoking, drug and alcohol use, and mood.	This change responded to focus group participants’ request for customization within the app.
Reminder messages and tailored feedback	Reminder messages and tailored feedback	Reminder messages were made optional. Tailored feedback was expanded to include messages on new tracking options (ie, exercise, smoking).	Focus group participants noted that reminders are an important feature to include in an adherence app. However, those who already have strategies for remembering to take their medications may not need them. The change allows users to customize the app to their needs. Expansion of tailored feedback was designed to provide additional customized feedback on factors related to adherence.
Friends	Allies	The name was changed from “Friends” to “Allies”. The feature was expanded to include the “Spotlight” feature described in the Battle section above.	The name was changed to align with the storyline and superhero theme. The “Spotlight” feature enhanced the ability for users to interact with their Allies in an interactive and fun way.
Information modules	Daily Dose	The information modules were replaced with the Daily Dose, an app newspaper that follows a curriculum of daily short articles and tips to address HIV and ART knowledge and promote disease management. Reading an article earns users points that can be used to buy new cards for the Battle.	The Daily Dose replaced the educational modules so that informational needs could be addressed in an engaging and interactive manner (ie, proper dosing guidelines, common medication side effects, coping with side effects, HIV acceptance process, identifying sources of social support). Users are encouraged to log into the app daily to get a new article. Awarding points increases motivation for reading articles and app engagement.
Gamification	Gamification	The gamification principles were expanded so that users earned points for each completed activity in the app. A “leveling up” feature was added so that users could unlock new battles.	The additional gamification features were added to increase motivation for behavior change and app use.

**App Storyline.**

The year is 2024, a pharmaceutical plant has just exploded sending poisonous rays throughout the city of Medopolis. Walter, the scientist responsible for the accident, has managed to escape, though not without sustaining some damage. The poison has wreaked havoc in Medopolis and unleashed monsters who are trying to destroy the city. Only you and your superhero allies can help. Walter will be your guide as you work together to gain knowledge and power to fight the monsters and restore Medopolis to its former glory.

#### Usability Testing Results

The mean and median age of participants was 23 years (n=7). Six participants identified as black or African American and one identified as white. Over half (4/7) earned less than US$20,999 per year. All participants owned a smartphone and more than half (4/7) had used a health app in the past 3 months. All participants were on ART to treat their HIV infection, but less than a third (2/7) said they never missed any of their medication.

**Figure 4:**
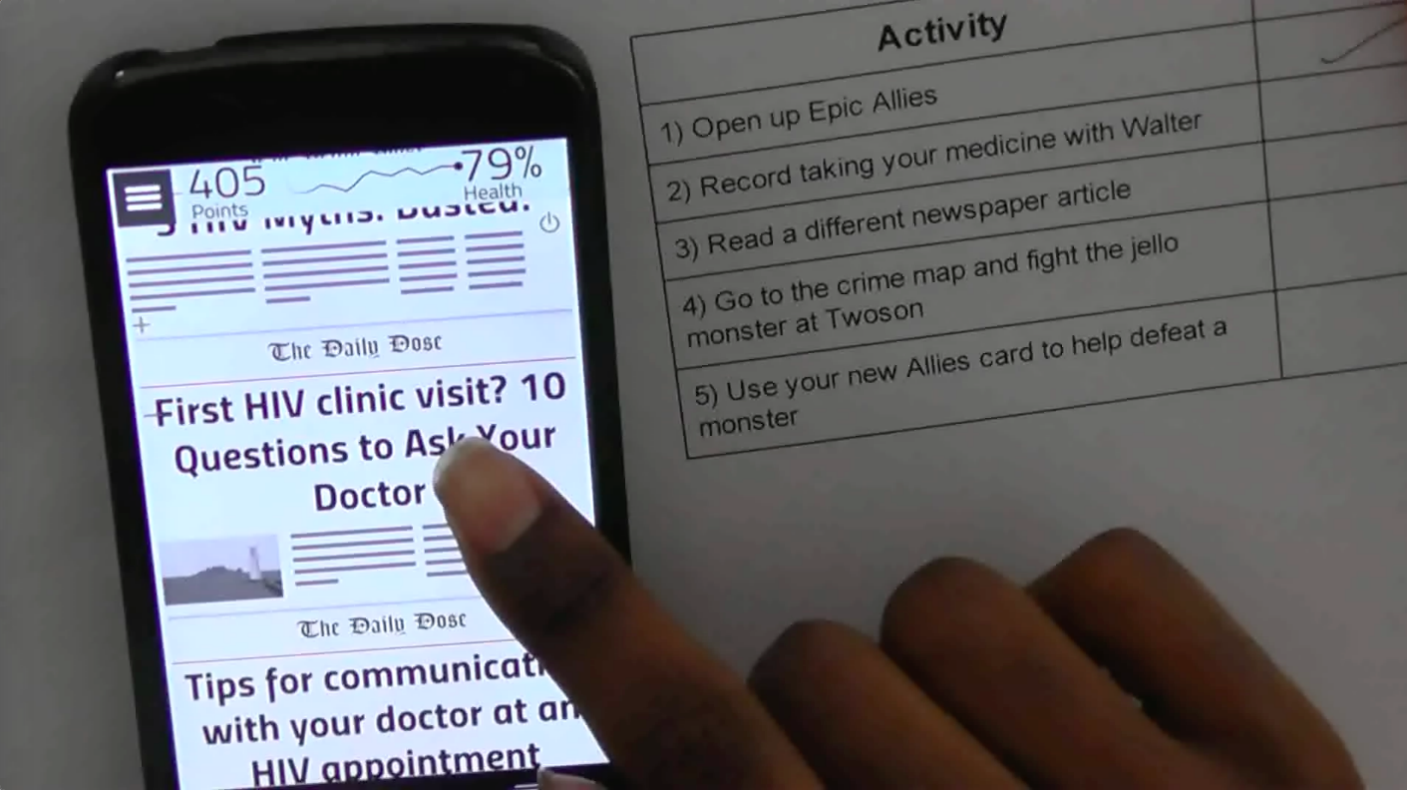
Usability testing with checklist.

Usability testing assessed participants’ ability to successfully navigate the app, comprehend the educational content, and determine if they found the app to be engaging and relevant. Usability scores are presented in Table 3. For all items, the mean responses indicated a favorable evaluation (agree/strongly agree) of Epic Allies. All participants were able to successfully complete the checklist of tasks representing days 1, 21, and 42 of app use. The checklist included tasks such as entering medications, setting up a medication reminder, reading the Daily Dose newspaper, upgrading Battle cards, playing Battles, and calling on an Ally to help them during a battle using the “Spotlight” card (Figure 4).

**Table 3:** Usability testing score means (n=7).

	Mean	Standard Deviation
Visually appealing	1.9	1.2
Overall impression is favorable	1.6	0.8
Medication tracking features easy to understand	1.7	1.1
Layout and structure easy to understand and navigate	2.0	1.0
Functions were easy to use	1.6	1.1
Interesting	1.3	0.5
Could help with medication taking	1.4	0.8
Can see the benefits of using an app like this	1.6	1.0
Could see myself using an app like this	1.7	1.0

^a^Score key: 1=strongly agree to 5=strongly disagree.

Overall, usability testing participants found the app to be engaging and relevant to their lives. As one man noted:

[I]t would be a game that I would play every day and it would make me, you know, it would make me want to join more programs like this to help others with HIV. And it would keep me on my medicine, keep exercising, and keep me motivated, whatever. I would be good. If I ever do get in the mood, I take this game out and play it. And, it’s fun. And it actually educates people that don’t know anything about HIV.

Participant 5

Participants found the informational content delivered through the Dashboard and the Daily Dose features informative and easy to comprehend. Remarking on the information provided through the Dashboard:

To be able to see and realize, ok well last month I had this much bad days and this much good days to be able to go back and think what was I doing last month comparatively this month, maybe thinking about why I’m having better days this month. Just being able to monitor it I guess and seeing the difference.

Participant 4

The fact that it monitors your mood, your medication, and your exercise is a really good thing. To be able to monitor and to be able to see a pattern of how your days usually go would make me feel a little bit better.”

Participant 2

Participants also commented on the Daily Dose content:

I thought [the Daily Dose articles] were all informative pieces and they definitely all hit things that [are important]… but looking back early in my diagnosis I can’t say I knew all of these things.

Participant 1

It’s something I can relate to, why not read up on it and understand more about something you’re living with. This is your life. You wanna be educated on as much as possible on what’s going on because this day and age things change. And it gets real really quick.

Participant 4

Several participants commented on the importance of the Allies social networking element. The overarching theme was that the feature could help participants feel they are not alone in taking medications for HIV.

The app gives you the sense that you aren’t alone. I’m not the only one keeping up with my meds everyday and playing the game everyday.

Participant 3

While most feedback about the app was positive, participants also made suggestions for app improvement. Some men felt the Battle feature of the app would be more engaging with increased interactivity. They expressed concerns about the turn-based mechanics in the card game:

Well, I don’t like the fact that the enemy just automatically ‘gets his turn’ and I just gotta stand back for the enemy to just demolish me. I would really like, if it’s possible, you know how like computer video games are…I guess more of an interactive fight.

Participant 3

After completing the tasks assigned for hypothetical Day 1 of app use, over half (4/7) of usability testing participants did not fully understand how the character in the Battle was associated with adherence. By the end of the usability session, all participants were able to describe the relationship between the Battle character and adherence. However, participants noted that it is important to clearly establish this link during the first day of app use.

## Discussion

### Overview

Smartphone app interventions are increasingly used to promote positive health behaviors among individuals living with HIV, including ART adherence [10,28-30]. In this study, a smartphone app was viewed as a highly acceptable tool for the delivery of an ART adherence intervention. Other studies have also found that adult men who have sex with men and YMSM view smartphone apps as an acceptable delivery mechanism for HIV-related interventions [31-34]. However, to our knowledge, no ART adherence apps have been designed to meet the unique adherence needs of YMSM. This study used an iterative development process to create a theory-based game adherence app for YMSM. Based on input and feedback from study participants, the initial app concept was incrementally modified and enhanced to create the final app prototype. This highlights the importance of seeking target population input and using an iterative process in the development of behavior change apps.

Focus group participants identified key IMB needs and proposed strategies for addressing these needs through a smartphone app. The men identified a need for specific information about adherence-related topics, such as dosing schedules and side effects, and recommended that the app provide general information about HIV and adherence. Medication side effects and side effects management was a specific area of interest because, as in other studies of HIV-positive youth side effects were identified as a barrier to adherence [35,36]. The Daily Dose feature, a virtual newspaper delivered through the app daily, was developed for the final prototype. The daily nature of the feature allowed for an increase in the amount of content delivered and provided an opportunity to address the specific and general informational needs identified in the focus groups.

A key theme associated with motivation for adherence was acceptance of HIV status. Several men noted that those who have not yet accepted their diagnosis might not be motivated to take their medications because doing so reminds them that they have HIV. Pill taking as a reminder of HIV infection has been found to be a key barrier to adherence in other studies of youth and young adults [35]. In contrast, social support was viewed as a motivating factor for adherence and participants recommended that the app include opportunities for users to give and receive social support. Among youth and young adults, social support has been identified as a facilitator for ART adherence while lack of support is a barrier [35,37]. In one study of racial and ethnic minority youth, greater social support predicted greater self-efficacy, which was associated with improved adherence [37]. In other studies, YMSM have indicated a preference for HIV-related apps that provide a platform for giving and receiving support [32,33]. Presenting opportunities for interactions with other HIV-positive YMSM through the Allies feature of Epic Allies may help those struggling with acceptance of HIV status feel like a part of a community and increase perceptions of support for adherence.

Consistent with other studies on ART adherence among youth and young adults [35,38], forgetting to take medications was identified as a challenge. Even though some men had developed strategies for remembering to take their medications and did not need reminders, participants agreed that reminders should be included in an ART adherence app. In response to the desire for customization, the medication reminder feature was made optional in the final prototype developed in Phase Three.

Participants also provided important guidance on app characteristics that would motivate regular and sustained app use. Interactivity through games, points, rewards, and social interactions were discussed as motivating factors for app use. Among adult and young MSM, interactivity has been emphasized as a necessary function of mobile apps [33,39]. The superhero theme and Battle feature introduced during the last focus group was viewed as a promising approach to further enhance app interactivity. While customization was viewed as important for meeting unique adherence needs, it was also important for motivation for app use. For example, participants noted that unnecessary reminders would be annoying and intrusive and increase the likelihood that they would discontinue app use. Another study of YMSM also found a preference for customization in HIV-related app interventions with unnecessary reminders and alerts resulting in app avoidance or deletion [33].

The feedback gathered during usability testing in Phase Three was generally positive and constructive and offered actionable guidance for changes to the app features prior to further testing. In response to suggestions provided during usability testing, the turn-based mechanics in the Battle were eliminated to increase interactivity and engagement. In order to quickly establish the link between real-world medication adherence and the character’s strength and performance in the game, the virtual guide’s initial app introduction was modified to explicitly explain the relationship. The Dashboard was changed so that it featured more prominently in the app and now serves as a constant reminder of the relationship between the user’s adherence behaviors and game play.

### Limitations

Our study was limited by its small sample size and geographical region. Though common in qualitative research, this limits the ability to generalize the study findings to all YMSM in the United States. Further, the majority of study participants identified as black or African American, which reflects the high burden of infection among black or African American individuals in study area [40]. While this may limit generalizability of findings to YMSM of other races or ethnicities, it should be noted that none of the app recommendations were unique to black MSM [39,41]. It is highly likely that the findings are relevant for YMSM of all races and ethnicities. However, future studies are needed to test the app with a more racially and ethnically diverse sample.

Epic Allies represents only one type of technology-based intervention that could be developed to address ART adherence among YMSM. Other less costly approaches could be used, such as interventions built on existing social media sites or adaption of currently available medication adherence apps for other medical conditions. However, our formative work with YMSM in the United States found a demand for an app intervention that integrates multiple features including behavior tracking, social networking features, and gaming features [32,33]. Existing commercial HIV apps often lack a theoretical basis, are limited in the topics they address and features they offer, have failed to capture the attention of end users and have not undergone rigorous evaluation [28,29]. If successful, the planned commercialization of Epic Allies would justify the costs invested and address the dearth of theoretically grounded, multicomponent commercial apps designed to address ART adherence.

The IMB is a well-established model of behavior change that has been used by researchers to guide the development of technology-based HIV interventions [42-44]. However, modifications of existing behavior change models or theories, including the IMB, are needed to more effectively account for the dynamic nature of technology-based interventions [45,46]. Such modifications will help guide future intervention development and evaluation of engagement and effectiveness [45,46]. With an eye toward adapting existing theories, our team has developed alternative measures of engagement in technology-based interventions and will be exploring these and other approaches in the randomized controlled trial of Epic Allies [47]. Furthermore, we will seek to identify the specific elements of Epic Allies that contribute to changes in information, motivation, and behavioral skills and, ultimately, medication adherence.

The results from usability testing indicated a positive response to the Epic Allies app. However, these findings were limited to a walk-through of the app in a controlled environment. This type of testing, while appropriate for app development, may not reveal barriers to implementation in the real world. The app was carefully designed to quickly engage users, sustain motivation for daily, long-term app use, and minimize the usage of phone data; however, the success of these strategies will not be known until the app is tested in a randomized controlled trial. In spite of these limitations, this study provides key insights into a process of app development that engages the target population to develop an app appropriate for end-user needs and preferences.

### Conclusion

Focus group and usability findings confirmed the appropriateness of a game-based app with social networking features to address ART adherence for YMSM. As evidenced by the changes made to the app between initial concept and final prototype, an iterative approach is critical for developing an app that is relevant, engaging and useful. This multistage process of development can be used to develop different types of health behavior apps for a wide range of populations.

## Abbreviations

ART: antiretroviral therapy
HIV: human immunodeficiency virus
UNC: University of North Carolina
YMSM: young men who have sex with men

## References

[1] Centers for Disease ControlPrevention (CDC) (2012). Vital signs: HIV infection, testing, and risk behaviors among youths - United States. MMWR Morb Mortal Wkly Rep.

[2] Johnson AS, Hall HI, Hu X, Lansky A, Holtgrave DR, Mermin J (2014). Trends in diagnoses of HIV infection in the United States, 2002-2011. JAMA.

[3] Zanoni BC, Mayer KH (2014). The adolescent and young adult HIV cascade of care in the United States: exaggerated health disparities. AIDS Patient Care STDS.

[4] Bangsberg DR, Perry S, Charlebois ED, Clark RA, Roberston M, Zolopa AR, Moss A (2001). Non-adherence to highly active antiretroviral therapy predicts progression to AIDS. AIDS.

[5] Haubrich RH, Little SJ, Currier JS, Forthal DN, Kemper CA, Beall GN, Johnson D, Dubé MP, Hwang JY, McCutchan JA (1999). The value of patient-reported adherence to antiretroviral therapy in predicting virologic and immunologic response. California Collaborative Treatment Group. AIDS.

[6] Mannheimer S, Friedland G, Matts J, Child C, Chesney M (2002). The consistency of adherence to antiretroviral therapy predicts biologic outcomes for human immunodeficiency virus-infected persons in clinical trials. Clin Infect Dis.

[7] Paterson DL, Swindells S, Mohr J, Brester M, Vergis EN, Squier C, Wagener MM, Singh N (2000). Adherence to protease inhibitor therapy and outcomes in patients with HIV infection. Ann Intern Med.

[8] Quinn TC, Wawer MJ, Sewankambo N, Serwadda D, Li C, Wabwire-Mangen F, Meehan MO, Lutalo T, Gray RH (2000). Viral load and heterosexual transmission of human immunodeficiency virus type 1. Rakai Project Study Group. N Engl J Med.

[9] Pew RC (2015). U.S. Smartphone use in 2015.

[10] Muessig KE, Nekkanti M, Bauermeister J, Bull S, Hightow-Weidman LB (2015). A systematic review of recent smartphone, internet and web 2.0 interventions to address the HIV continuum of care. Curr HIV/AIDS Rep.

[11] Kato PM (2010). Video games in health care: closing the gap. Review of General Psychology.

[12] Brown SJ, Lieberman DA, Germeny BA, Fan YC, Wilson DM, Pasta DJ (1997). Educational video game for juvenile diabetes: results of a controlled trial. Med Inform (Lond).

[13] Baranowski T, Baranowski J, Cullen KW, Marsh T, Islam N, Zakeri I, Honess-Morreale L, deMoor C (2003). Squire's Quest! Dietary outcome evaluation of a multimedia game. Am J Prev Med.

[14] Kato PM, Cole SW, Bradlyn AS, Pollock BH (2008). A video game improves behavioral outcomes in adolescents and young adults with cancer: a randomized trial. Pediatrics.

[15] Lieberman D, Street RL, Gold WR, Manning T (1997). Interactive video games for health promotion: effects on knowledge, self-efficacy, social support health. Health Promotion and Interactive Technology: Theoretical Applications and Future Directions.

[16] Balaji AB, Oster AM, Viall AH, Heffelfinger JD, Mena LA, Toledo CA (2012). Role flexing: how community, religion, and family shape the experiences of young black men who have sex with men. AIDS Patient Care STDS.

[17] Lewis LJ, Kertzner RM (2003). Toward improved interpretation and theory building of African American male sexualities. J Sex Res.

[18] Stokes JP, Peterson JL (1998). Homophobia, self-esteem, and risk for HIV among African American men who have sex with men. AIDS Educ Prev.

[19] Pew Internet American Life Project (2011). The Social Life of Health Information, 2011.

[20] Pew Research Center (2015). Social media usage, 2005-2015.

[21] Pew Research Center (2008). Adults and video games.

[22] Fisher JD, Fisher WA, Amico KR, Harman JJ (2006). An information-motivation-behavioral skills model of adherence to antiretroviral therapy. Health Psychol.

[23] (2015). Dedoose Version 6.1.18, web application for managing, analyzing, and presenting qualitative and mixed method research data.

[24] Lewis J, Sauro J (2009). The factor structure of the System Usability Scale. Human Centered Design: Lecture Notes in Computer Science.

[25] Lewis JR (1995). IBM computer usability satisfaction questionnaires: psychometric evaluation and instructions for use. International Journal of Human-Computer Interaction.

[26] Coursaris C, Kim D (2011). A meta-analytical review of empirical mobile usability studies. Journal of Usability Studies.

[27] Borsci S, Federici S, Lauriola M (2009). On the dimensionality of the System Usability Scale: a test of alternative measurement models. Cogn Process.

[28] Hightow-Weidman LB, Muessig KE, Bauermeister J, Zhang C, LeGrand S (2015). Youth, technology, and HIV: recent advances and future directions. Curr HIV/AIDS Rep.

[29] Muessig KE, Pike EC, Legrand S, Hightow-Weidman LB (2013). Mobile phone applications for the care and prevention of HIV and other sexually transmitted diseases: a review. J Med Internet Res.

[30] Sullivan PS, Jones J, Kishore N, Stephenson R (2015). The roles of technology in primary HIV prevention for men who have sex with men. Curr HIV/AIDS Rep.

[31] Levy ME, Watson CC, Wilton L, Criss V, Kuo I, Glick SN, Brewer RA, Magnus M (2015). Acceptability of a mobile smartphone application intervention to improve access to HIV prevention and care services for Black men who have sex with men in the District of Columbia. Digit Cult Educ.

[32] LeGrand S, Muessig KE, Pike EC, Baltierra N, Hightow-Weidman LB (2014). If you build it will they come? Addressing social isolation within a technology-based HIV intervention for young black men who have sex with men. AIDS Care.

[33] Muessig KE, Pike EC, Fowler B, LeGrand S, Parsons JT, Bull SS, Wilson PA, Wohl DA, Hightow-Weidman LB (2013). Putting prevention in their pockets: developing mobile phone-based HIV interventions for black men who have sex with men. AIDS Patient Care STDS.

[34] Holloway IW, Rice E, Gibbs J, Winetrobe H, Dunlap S, Rhoades H (2014). Acceptability of smartphone application-based HIV prevention among young men who have sex with men. AIDS Behav.

[35] MacDonell K, Naar-King S, Huszti H, Belzer M (2013). Barriers to medication adherence in behaviorally and perinatally infected youth living with HIV. AIDS Behav.

[36] Macdonell KE, Naar-King S, Murphy DA, Parsons JT, Huszti H (2011). Situational temptation for HIV medication adherence in high-risk youth. AIDS Patient Care STDS.

[37] Kolmodin MK, Naar S, Isabella FM, ATN 086/106 Protocol Team (2015). Predictors of self-reported adherence to antiretroviral medication in a multisite study of ethnic and racial minority HIV-positive youth. J Pediatr Psychol.

[38] Murphy DA, Sarr M, Durako SJ, Moscicki A, Wilson CM, Muenz LR, Adolescent Medicine HIV/AIDS Research Network (2003). Barriers to HAART adherence among human immunodeficiency virus-infected adolescents. Arch Pediatr Adolesc Med.

[39] Goldenberg T, McDougal SJ, Sullivan PS, Stekler JD, Stephenson R (2014). Preferences for a mobile HIV prevention app for men who have sex with men. JMIR Mhealth Uhealth.

[40] North Carolina HIV/STD Surveillance Unit (2015). Raleigh, North Carolina: North Carolina Department of Health and Human Service.

[41] Goldenberg T, McDougal S, Sullivan P, Stekler J, Stephenson R (2015). Building a mobile HIV prevention app for men who have sex with men: an iterative and community-driven process. JMIR Public Health Surveill.

[42] Fisher JD, Amico KR, Fisher WA, Cornman DH, Shuper PA, Trayling C, Redding C, Barta W, Lemieux AF, Altice FL, Dieckhaus K, Friedland G, LifeWindows T (2011). Computer-based intervention in HIV clinical care setting improves antiretroviral adherence: the LifeWindows Project. AIDS Behav.

[43] Mustanski B, Garofalo R, Monahan C, Gratzer B, Andrews R (2013). Feasibility, acceptability, and preliminary efficacy of an online HIV prevention program for diverse young men who have sex with men: the keep it up! intervention. AIDS Behav.

[44] Aliabadi N, Carballo-Dieguez A, Bakken S, Rojas M, Brown W, Carry M, Mosley JP, Gelaude D, Schnall R (2015). Using the Information-Motivation-Behavioral Skills Model to guide the development of an HIV prevention smartphone application for high-risk MSM. AIDS Educ Prev.

[45] Allison S, Bauermeister JA, Bull S, Lightfoot M, Mustanski B, Shegog R, Levine D (2012). The intersection of youth, technology, and new media with sexual health: moving the research agenda forward. J Adolesc Health.

[46] Riley WT, Rivera DE, Atienza AA, Nilsen W, Allison SM, Mermelstein R (2011). Health behavior models in the age of mobile interventions: are our theories up to the task?. Transl Behav Med.

[47] Baltierra NB, Muessig KE, Pike EC, LeGrand S, Bull SS, Hightow-Weidman LB (2016). More than just tracking time: complex measures of user engagement with an internet-based health promotion intervention. J Biomed Inform.

